# Development of a Scoring System to Predict the Treatment Success for Nonoperative Management of Peptic Ulcer Perforation: A Secondary Data Analysis of PPAP Study

**DOI:** 10.1002/ags3.70074

**Published:** 2025-08-12

**Authors:** Kei Ito, Akira Endo, Hiromasa Hoshi, Koji Ito, Tomohiro Akutsu, Hikaru Odera, Hideto Shiraki, Takeshi Yokoyama, Yasukazu Narita, Taro Masuda, Akira Suekane, Shigeru Yamagishi, Koji Morishita

**Affiliations:** ^1^ Department of Acute Care Surgery Fujisawa City Hospital Fujisawa Japan; ^2^ Department of Acute Critical Care Medicine Tsuchiura Kyodo General Hospital Tsuchiura Japan; ^3^ Department of Gastroenterological Surgery Tsuchiura Kyodo General Hospital Tsuchiura Japan; ^4^ Department of Acute Critical Care and Disaster Medicine Institute of Science Tokyo Meguro Japan; ^5^ Department of Surgery Chiba Rosai Hospital Chiba Japan; ^6^ Department of Surgery Ohtanishinouchi General Hospital Koriyama Japan; ^7^ Department of Surgery Mito Medical Center Higashiibaraki Japan; ^8^ Department of Emergency and Critical Care Center Matsudo City General Hospital Matsudo Japan; ^9^ Department of Surgery Fujisawa City Hospital Fujisawa Japan

**Keywords:** general surgery, nonoperative management, peptic ulcer perforation

## Abstract

**Background:**

Although surgical treatment is the primary measure for patients with perforated peptic ulcer (PPU), nonoperative management (NOM) has become a common alternative. However, risk score models predicting the success of NOM based on the analysis of a large number of patients remain scarce. We developed a clinically applicable scoring system to predict the success of NOM in patients with PPU using data from a large cohort.

**Method:**

We analyzed data of the Perforated Peptic ulcer Analyzing Project (PPAP), which was a retrospective survey of adult patients with PPU between January 2011 to December 2022. The successful NOM case was defined as patients who survived until hospital discharge without requiring surgery. Factors associated with NOM were identified using a multivariable logistic regression analysis, and a scoring system to predict NOM was developed by weighting these factors based on the regression coefficients.

**Result:**

Of 702 potentially eligible patients, 584 were treated with NOM, of which 130 patients (22.2%) were treated successfully. Age, sex, body temperature, heart rate, the extent of peritoneal irritation signs, C reactive protein, spread of ascites, and sepsis were included in the final model. Using these variables, we developed the scoring system named PPAP score, which had favorable discriminating ability with the area under receiving operating characteristic curve of 0.799. When the cut‐off was set to 56, the sensitivity and the specificity were 0.738 and 0.722, respectively.

**Conclusion:**

A predictive scoring model was developed. However, external validation of the model is required to confirm its clinical applicability.

## Abstract 2

This multicenter retrospective study developed a scoring system (PPAP score) to predict the success of nonoperative management (NOM) in patients with perforated peptic ulcer. The model, based on clinical data at diagnosis, showed good predictive performance with an AUROC of 0.799.

**List of Abbreviations**
Alb: albumin
AUROC: area under the receiver operating characteristic curve
BT: body temperature
CCI: Charlson Comorbidity index
Cre: creatinine
CRP: C reactive protein
CT: computed tomography
Hb: hemoglobin
HR: heart rate
NOM: nonoperative management
PPU: perforated peptic ulcer
RCT: randomized controlled trial
sBP: systolic blood pressure
WBC: white blood cell counts

## 1 Background

Although the global prevalence of peptic ulcer disease has been declining, it remains a significant cause of hospital admissions worldwide [1]. The perforated peptic ulcer (PPU) is a medical emergency with reported mortality rates ranging from 10%–30% [2, 3]. Although surgical treatment is the standard therapy for patients with PPU, nonoperative management (NOM) has also been an alternative owing to the spread of proton pump inhibitors [4, 5, 6, 7]. Notably, no study has reported any significant difference in mortality between surgical treatment and NOM [8]. The advantages of NOM include avoiding surgical invasiveness and potentially reducing surgical resource utilization. While the rate of clinical improvement and achievement for NOM in PPU cases is approximately 54%, some cases result in the failure of NOM. Given that the mortality rate increases with every hour of delay to surgery, NOM should be carefully considered [4]. The risk factors for the success of NOM include the size of the pneumoperitoneum, heart rate, abdominal meteorism, and low serum albumin levels [9, 10]. However, these studies were single‐center retrospective studies, highlighting the need for a study with larger sample sizes.

Although risk scoring models such as the Boey and PULP scores have been developed [11, 12] for surgical treatment, only a few studies [13, 14] have developed score models to predict the success of NOM. Therefore, in this study, we aimed to develop a clinical score to predict the success of NOM using data from a multicenter study.

## 2 Methods

### 2.1 Study Design and Setting

We analyzed data from the Perforated Peptic ulcer Analyzing Project (PPAP) [15]. The study was a retrospective observational study conducted at seven centers in Japan from January 2011 to December 2022. All centers are high‐volume centers with expertise in emergency surgery, performing over 1000 surgeries per year, and all surgeries were generally performed by either gastroenterological surgeons or acute care surgeons. The PPAP included consecutive patients aged 18 years or older who were diagnosed with PPU. We excluded pregnant women, patients who refused standard treatment including surgery, patients with malignancy‐related or iatrogenic perforation, patients who opted out of their clinical data being used, and patients who were judged as inappropriate for the study by an investigator at each facility. The details of the PPAP study were published elsewhere [15]. This study complied with the principles of the 1964 Declaration of Helsinki and its later amendments. This study was approved by the institutional review board of the principal institute (2023FY181). As this study was an observational study rather than a clinical trial, registration in a clinical trial registry was not required. The study was reported in accordance with the Transparent Reporting of a Multivariable Prediction Model for Individual Prognosis or Diagnosis reporting guideline.

### 2.2 Study Population

Eligible patients of this study were those diagnosed with PPU by computed tomography (CT) at the initial visit. The PPU includes both gastric and duodenal PPU. The cases with missing values were excluded from the analysis.

### 2.3 Data Collection

The following patient data were obtained from the PPAP database: age, sex, history of medication of non‐steroidal anti‐inflammatory drugs and steroid, body temperature (BT), systolic blood pressure (sBP), heart rate (HR), white blood cell counts (WBC), C reactive protein (CRP), hemoglobin (Hb), albumin (Alb), creatinine (Cre), Charlson comorbidity index (CCI), presence or absence of sepsis at the diagnosis, time from onset to hospital visit, extent of peritoneal irritation signs, extent of ascites in CT, hospital length of stay, in‐hospital mortality, and successful completion of NOM.

### 2.4 Definitions and Outcome Measures

The successful NOM case was defined as patients who survived until hospital discharge without requiring surgery; thus, the remaining population presented patients who required surgery upon hospital admission or after attempting of NOM, or patients who died following NOM. Notably, patients who developed complications such as intra‐abdominal abscesses and subsequently required percutaneous drainage, but did not undergo surgical intervention, were still considered to have achieved successful NOM. Time from onset to hospital visit was classified into four categories based on the time: < 6 h, 6–12 h, 12–24 h, and more than 24 h. Peritoneal irritation signs were classified into three categories based on their spread: none, upper abdomen only, and wider than upper abdomen. Spread of ascites was assessed by CT findings and classified into three categories based on its spread: none, upper abdomen only, and wider than upper abdomen. Vital signs and blood test data were collected at the time of diagnosis. All PPU cases were diagnosed by CT findings. Sepsis and septic shock were defined according to the Sepsis‐3 criteria [16]. Primary outcome was the success of NOM.

### 2.5 Statistical Analysis

The enrolled patients were categorized into two groups according to the completion of NOM or not. We conducted a univariate analysis using Student's *t*‐test or the Mann–Whitney U‐test to compare continuous variables. We also used the χ^2^ test or Fisher's exact test to compare categorical variables, as appropriate. Also, a multivariable logistic regression analysis was performed for the development of the prediction model for the success of NOM. The variables used in the prediction model were selected based on the subject matter knowledge and the risk factors for morbidity and mortality in previous studies [8, 9, 12, 13]. Variables with a *p*‐value < 0.2 in the univariate analysis were also incorporated into the multivariate model. Multicollinearity among variables was assessed using variance inflation factor (VIF), with a threshold of VIF < 2 considered acceptable. Variable selection for the final model was performed using stepwise regression with the minimum Akaike's Information Criterion, and the discriminative ability was evaluated using the area under the receiver operating characteristic curve (AUROC). In this variable selection step, we conducted internal validation using bootstrapping (500 iterations) to evaluate the stability and estimate optimism of the multivariable logistic regression model. For each resampled dataset with allowed duplicates, we repeated the full modeling procedure, including stepwise variable selection based on AIC, and constructed a multivariable logistic regression model. The AUROC was calculated for both the bootstrap model applied to the bootstrap sample and the same model applied to the original dataset. The difference between these AUROCs was defined as optimism, and the average of these differences across 500 iterations was taken as the overall optimism estimate in this study. To evaluate the calibration of the model, we constructed a calibration plot and performed the Hosmer–Lemeshow test.

Continuous variables in the multivariable logistic regression analysis were first converted into ordinal categories based on clinically meaningful thresholds. Subsequently, we re‐performed multivariable logistic regression using these variables to construct the scoring system. Each variable was subsequently assigned a weighted score in integer form, using the smallest regression coefficient as the reference (i.e., assigned a score of 1), with other variables scaled proportionally to reflect their relative strength of association. The cut‐off value was calculated as the nearest integer to the point where the Youden index reached its maximum. Sample size estimation and validation cohort analysis were not performed.

All statistical analyses were performed using R software (version 4.4.0; R Foundation for Statistical Computing, Vienna, Austria) and a commander module incorporating frequently used biostatistical functions. Differences were considered statistically significant at two‐sided *p*‐values of < 0.05.

## 3 Results

**FIGURE 1:**
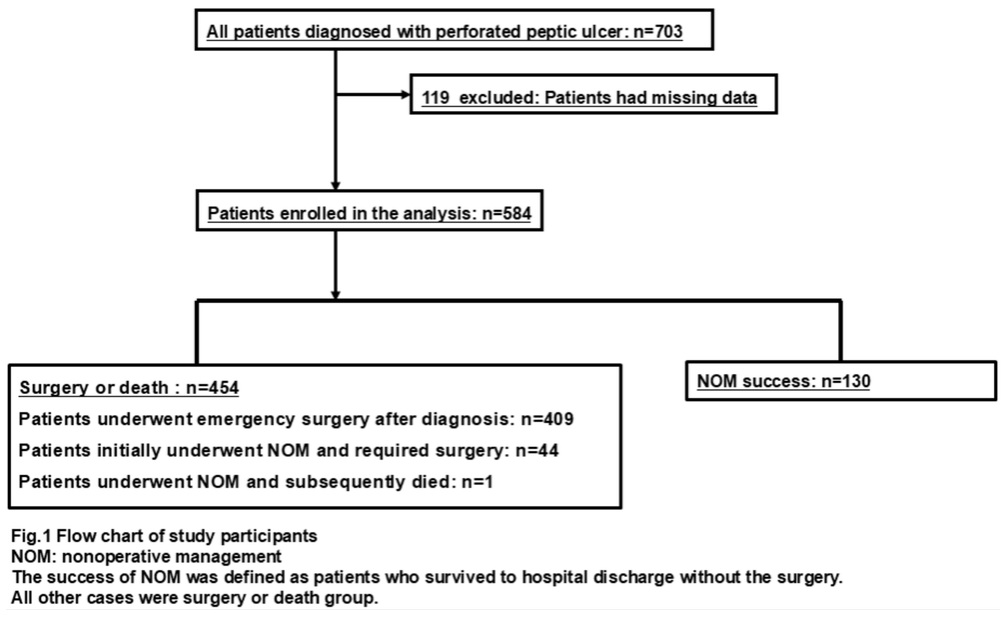
Flow chart of study participants. NOM, Nonoperative management. Success of NOM was defined as patients who survived to hospital discharge without the surgery. All other cases were defined as the failure of NOM.

Of 703 potentially eligible patients with PPU, 584 were included in the analysis (Figure 1). NOM was initially attempted in 174 cases. Of these, 130 patients (22%) successfully completed NOM, while 44 initially underwent NOM and subsequently required surgical intervention. The remaining 409 patients underwent emergency surgery at the time of initial presentation without an attempt at NOM. One patient underwent NOM and died. The baseline characteristics of patients and outcomes according to the success of NOM or not are presented in Table 1. In baseline characteristics, the statistically significant difference between the two groups was observed in the age, BT, HR, peritoneal irritation signs, CRP, Alb, Cre, spread of ascites, sepsis, and CCI. Regarding patient outcome, the length of hospital stay was 18 days in the NOM success group and 24 days in the remaining patients (i.e., emergency surgery or NOM failure). In‐hospital mortality was not observed in the NOM success group and 27 cases (6.0%) in the other patients. Extensive ascites was observed in CT findings among 84% of patients who died after surgery (Table S1).

**TABLE 1:** The baseline characteristics of patients.

Variables	Surgery or death *n* = 454^b^	Success *n* = 130^b^	*p* ^c^
Sex, female	133 (29.3)	50 (38.5)	0.047
Age	63 (17)	59 (17)	0.009
Non‐steroidal anti‐inflammatory drugs	96 (21.1)	27 (20.8)	0.926
Steroid	16 (3.5)	3 (2.3)	0.779
Charlson comorbidity index	5 (2)	4 (2)	0.026
Peritoneal_irritation_signs			< 0.001
None	55 (12.1)	40 (30.8)	
Upper abdomen only	153 (33.7)	69 (53.1)	
Wider than upper abdomen	246 (54.2)	21 (16.2)	
Ascites in CT			< 0.001
None	65 (14.3)	45 (34.6)	
Upper abdomen only	70 (15.4)	29 (22.3)	
Wider than upper abdomen	319 (70.3)	56 (43.1)	
Body temperature, °C	36.8 (0.85)	36.9 (0.75)	0.028
Systolic blood pressure, mmHg	133 (28)	134 (26)	0.696
Heart Rate, bpm	97 (22)	88 (17)	< 0.001
White blood cell count, /μL	12 332 (9032)	12 314 (9097)	0.664
C reactive protein, mg/dL	7 (11)	4 (7)	0.001
Hemoglobin, mg/dL	13.1 (3.4)	12.9 (3.1)	0.596
Albumin, g/dL	3.5 (1.49)	3.8 (0.69)	< 0.001
Serum creatinine, mg/dL	1.26 (1.40)	0.92 (0.64)	0.011
Sepsis			< 0.001
None	398 (87.7)	129 (99.2)	
Sepsis	29 (6.3)	1 (0.8)	
Septic shock	27 (6.0)	0 (0.0)	
Time from onset to hospital visit, hours			0.194
Less than 6	200 (44.1)	70 (53.8)	
6–12	96 (21.1)	21 (16.2)	
12–24	56 (12.3)	11 (8.5)	
More than 24	102 (22.5)	28 (21.5)	
Perforated_area^a^			< 0.001
Anterior wall of the stomach	156 (34.4)	21 (16.2)	
Posterior wall of the stomach	23 (5.1)	10 (7.7)	
Anterior wall of the duodenum	255 (56.2)	46 (35.4)	
Posterior wall of the duodenum	16 (3.5)	4 (3.1)	
Unknown	4 (0.9)	49 (37.7)	
Perforation diameter, mm^a^	11 (10.4)	NA	NA
Hospital length of stay, days	24 (24)	18 (18)	0.002
In‐hospital mortality	27 (5.95)	0 (0.0)	0.004

*Note:* The definition of sepsis and septic shock is based on the Sepsis‐3 criteria.

Abbreviation: CT, computed tomography.

^a^ These data were not used in the development of the model.

^b^ *n* (%); Mean (SD); *n* (%).

^c^ Pearson's Chi‐squared test; Wilcoxon rank sum test.

In the multivariable analysis, age, sex, steroid use, CCI, BT, sBP, HR, peritoneal irritation signs, WBC, CRP, Alb, Cre, ascites in CT, sepsis, and time from onset to hospital arrival were included in the first model from univariate analysis. Given that age and CCI had VIF values > 2, considering the issue of multicollinearity, the CCI was removed from the model. Subsequently, all the VIF values of the variables were < 2.

Table 2 shows the results of multivariable analyses for the success of NOM. As a result, sex, age, BT, HR, peritoneal irritation signs, CRP, ascites, and sepsis were included in the final model. In the score development step, continuous variables were first converted into ordinal variables with reference to the cut‐off values in previous studies [17, 18, 19, 20]. Considering the usability in clinical settings, the cut‐off values were determined to provide approximately five categories per variable, simulataneously considering clinically meaningful upper and lower limits. Age was converted into an ordinal variable in increments of 20 years with reference to the age‐ajusted CCI [17], with a cut‐off of 80 years. Based on this, we divided the age range into five categories to ensure comprehensive coverage across all ages. BT was converted into an ordinal variable in increments of 0.5°C, with a basedline value of 36.5°C [18], to create five clinically meaningful categories. HR was converted into an ordinal variable in increments of 20 bpm, centered around 120 bpm [18], as wider intervals (e.g., 30 bpm) would unnecessarily assign points to extreme bradycardia. CRP was converted into an ordinal variable in increments of 10 mg/dL, with a baseline of 10 mg/dL [20] and maximum value of 30 mg/dL, as higher levels were rare. The multivariable analysis was re‐performed based on these ordinal variables (Table 3). To assign integer points, the smallest absolute coefficient in the model, which was that of BT (0.139 per 0.5°C increment), was selected as the reference. All coefficients were divided by this reference coefficient, and the resulting ratios were rounded to the nearest integer to simplify the score. For example, the coefficient for CRP level was 0.451 per 10 increment. When divided by the reference coefficient of 0.139, this yielded a ratio of approximately 3.24, which was rounded to 3. Therefore, 3 points were assigned for each 10 increase in the CRP level. The scoring system to predict success for NOM, named PPAP score, was developed (Table 4). The weight for each score was determined based on the regression coefficients of the model.

**TABLE 2:** Result of the logistic regression analysis for the success of NOM.

Characteristic	Regression coefficient	OR	95% CI	*p*
Sex, female	0.872	2.39	1.44, 4.01	0.001
Age	−0.017	0.983	0.970, 0.997	0.018
Body temperature	0.217	1.24	0.932, 1.66	0.139
Heart rate	−0.015	0.985	0.972, 0.996	0.013
Range of peritoneal signs	−1.00	0.366	0.264, 0.502	0.000
CRP	−0.034	0.966	0.935, 0.994	0.026
Ascites in CT	−0.397	0.673	0.516, 0.877	0.003
Sepsis	−1.93	0.145	0.009, 0.559	0.041

*Note:* Sex was treated as a binary variable. Female were coded as 1 and male as 0. Age, body temperature, heart rate, and CRP were treated as continuous variables. Range of peritoneal signs, ascites in CT, and sepsis were treated as ordinal variables. Peritoneal irritation signs were categorized as an ordinal variable with three categories: 0 for “None”, 1 for “Upper abdomen only”, and 2 for “Wider than upper abdomen”. Ascites in CT was classified as an ordinal variable with three categories: 0 indicating “None”, 1 indicating “Upper abdomen only”, and 2 indicating “Wider than upper abdomen”. Sepsis was categorized as an ordinal variable with three categories: 0 for “None”, 1 for “Sepsis”, and 2 for “Septic shock”, based on the Sepsis‐3 criteria.

Abbreviations: CI, confidence interval; CRP, C reactive protein; CT, computed tomography; OR, odds ratio.

**TABLE 3:** Result of the logistic regression analysis for success of NOM.

Characteristic	Regression coefficient	OR	95% CI	*p*
Sex (female)	0.784	2.19	1.32, 3.64	0.002
Age	0.264	1.30	1.00, 1.69	0.049
Body temperature	0.139	0.87	0.73, 1.03	0.113
Heart rate	0.272	1.31	1.04, 1.67	0.022
Range of peritoneal signs	0.993	2.70	1.97, 3.73	0.000
CRP	0.451	1.57	1.10, 2.38	0.021
Ascites in CT	0.395	1.48	1.14, 1.93	0.003
Sepsis	1.90	6.71	1.78, 110	0.042

*Note:* Sex was treated as a binary variable. Female ware coded as 1 and male as 0. The others were treated as ordinal variable. Age was converted into an ordinal variable in increments of 20 year. Body temperature was converted into an ordinal variable by each in increments of 0.5°C. Heart rate was converted into an ordinal variable by each in increments of 20 bpm. CRP was converted into an ordinal variable in increments of 10. Peritoneal irritation signs were categorized as an ordinal variable with three categories: 0 for “None”, 1 for “Upper abdomen only”, and 2 for “Wider than upper abdomen”. Ascites in CT was classified as an ordinal variable with three categories: 0 indicating “None”, 1 indicating “Upper abdomen only”, and 2 indicating “Wider than upper abdomen”. Sepsis was categorized as an ordinal variable with three categories: 0 for “None”, 1 for “Sepsis”, and 2 for “Septic shock”.

Abbreviations: CI, confidence interval; CRP, C reactive protein; CT, computed tomography; OR, odds ratio.

**TABLE 4:** The scoring system to predict success for NOM.

Variables	No. of points for NOM
Sex	
Female	6
Male	0
Age group, years	
Less than 20	8
20–40	6
40–60	4
60–80	2
80 and above	0
Body temperature, °C	
38 and above	4
37.5–38	3
37.0–37.5	2
36.5–37.0	1
Less than 36.5	0
Heart rate, bpm	
Less than 60	10
60–80	8
80–100	6
100–120	4
120–140	2
140 and above	0
Peritoneal irritation signs	
None	14
Upper abdomen	7
Wider than upper abdomen	0
CRP, mg/dL	
Less than 10	9
10–20	6
20–30	3
30 and above	0
Ascites in CT	
None	6
Upper abdomen	3
Wider than upper abdomen	0
Sepsis	
None	28
Sepsis	14
Septic shock	0

*Note:* The definition of sepsis and septic shock is based on the Sepsis‐3 criteria. The score ranges from 0 to 85 points. A score of 56 or higher was associated with a successful NOM, with a sensitivity of 0.738 and a specificity of 0.722.

Abbreviations: CRP, C reactive protein; CT, computed tomography; NOM, nonoperative management.

**FIGURE 2:**
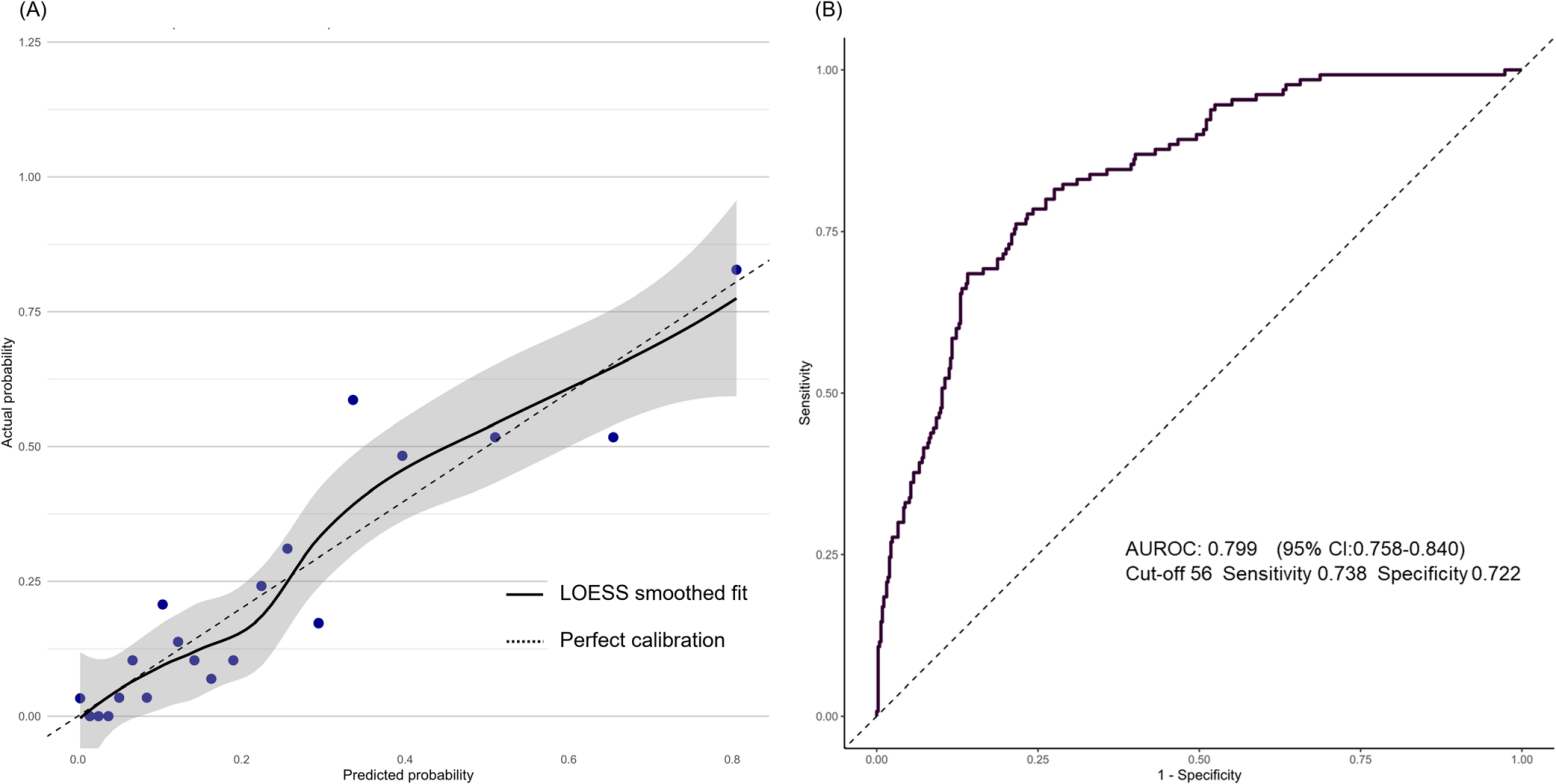
(A) The Calibraton plot of the multivariable model. The calibraiont plot indicated a good fit of the model. Hosmer–Lemeshow test indicated a good fit of the model (*χ* ^2^ = 8.35, *p* = 0.400). (B) The receiver operating characteristic curve of PPAP score for the successful NOM. A score of 56 or higher was associated with a successful NOM, with a sensitivity of 0.738 and a specificity of 0.713. AUROC, the area under the receiver operating characteristic curve; NOM, nonoperative management; ROC, the receiver operating characteristic curve.

The optimistic AUROC of this multivariable logistic model was 0.806 (95% confidence interval [CI]: 0.767–0.845), and the optimism‐corrected AUROC was 0.782. The calibration plot (Figure 2A) and Hosmer–Lemeshow test of the multivariable logistic model indicated a good fit of the model (*χ* ^2^ = 8.35, *p* = 0.400). Figure 2B shows the result of the ROC of PPAP score. The cutoff value of PPAP score was 56, sensitivity was 0.738, specificity was 0.722, and AUROC was 0.799 (95% CI: 0.758–0.840). The Hosmer–Lemeshow test indicated a good fit of the model (*χ* ^2^ = 0.509, *p* = 0.748).

## 4 Discussion

In this retrospective multicenter observational study, we developed a scoring system to predict successful NOM in patients with PPU. The value of AUROC of PPAP score was 0.798, which had sufficient discriminative ability for deciding NOM in clinical settings. To the best of our knowledge, this study is the largest‐scale report to investigated the success or failure of NOM for PPU.

Recognizing the appropriate patients for NOM in patients with PPU at the time of diagnosis may contribute to improving the prognosis of patients with PPU. While NOM allows the avoidance of surgical risks and reduces the invasiveness for the patients [4], careful attention should be given to any delays in surgical treatment considering the increasing mortality risk over time. Few studies revealed the risk for success or failure of NOM. Moreover, due to the differences in comparison groups, these studies did not always explore factors at the time of diagnosis [7, 9, 10]. In this study, patients who were diagnosed with PPU upon hospital arrival, regardless of whether the surgery was performed, were included. Additionally, detailed information such as the extent of peritoneal irritation signs and ascites, which was not contained in surgery databases in general but essential in clinical situations, was considered. No well‐designed randomized controlled trial (RCT) has compared the outcomes of surgical treatment and NOM. One possible reason is the lack of an established standard, making it challenging to objectively assess the common support region for surgery and NOM. The PPAP score developed in this study can aid in selecting appropriate candidates who are eligible for future RCTs, and support clinical decision‐making.

Owing to the aforementioned differences in methodology, the factors in the PPAP score were different from the factors used in the previous study. Age was reported in the previous study to be a risk for postoperative mortality and morbidity in patients with PPU [21, 22], and the spread of peritoneal irritation signs was also included as a factor of the Mannheim Peritonitis Index [23]. Regarding sex, the conflicting reports coexisted in previous studies [24]. In this study, the female sex was the independent factor in the success of NOM. The sensitivity of parietal cells to gastrin in females was lower than in males [25], and estrogen enhanced the mucosal protective effect in the duodenum [26]. This mechanism against gastric acid might explain the differences according to sex. The positive correlation between BT and the success of NOM could be affected by the severity of sepsis, in which hypothermia was reported to be a poor prognostic factor [27, 28].

In the present study, we did not consider the location and the size of the perforation. In surgical management, a previous study indicated that a larger perforation size or the presence of posterior wall ulcer was associated with a high conversion rate from laparoscopic surgery to open [29, 30]. However, since the location and the size of the perforation are generally confirmed by operative findings, incorporating them into the model established using the information at the diagnosis was inappropriate. For reference, the established model demonstrated reasonable predictive ability in subgroups based on the perforated organ (Figure S1). Furthermore, cases associated with malignancy were excluded from the analysis. In malignant cases, the probability of successful NOM is considered low, although no prior studies have specifically evaluated the prognosis of patients with perforated gastric cancer treated with NOM. It has been reported that perforation tends to occur in the advanced gastric cancer, where the disease has already progressed [31, 32], and there is concern of dissemination leading to poor prognosis.

It was reported that the median length of hospital stays in patients with PPU who received emergency surgery was 8.4 days [33] and that in patients who received NOM was 10–12 days [9]. Although the failure of NOM may lead to prolonged hospital stay, in this study, the length of hospital stays was 18 days, even in successful NOM cases. The length of hospital stays in Japan was reported to be longer compared to other countries in various diseases such as myocardial infarction and rectal cancer [34, 35]; however, it was reported that the mortality rate does not change even with an extended length of hospital stay [35]. Non‐clinical factors, including the unique Japanese insurance system, may have contributed to the prolonged length of hospital stay.

The strength of our study is that we used the PPU‐specific data from a multicenter, which included more detailed information compared to general databases, such as the range of peritoneal irritation signs and the number of ascites. The model was established based on the appropriate statistical approach, and the results were interpretable. However, this study had limitations. The issue of residual confounding could not be avoided due to the retrospective nature of the study. Some clicnical data, such as the readmission rate in NOM and the amount of pneumoperitoneum on CT, were not collected. The initial decision for NOM was not standardized and was left at the discretion of the physicians at each facility. Additionally, the treatment of NOM was not protocolized, resulting in variation in clinical practices between facilities. The cross‐validation could not be performed due to the limited sample size. Despite analyzing a larger dataset compared to previous studies, generalizability was limited since all data were from limited hospitals in the country.

## 5 Conclusion

We developed a scoring system to predict successful NOM for PPU. Further external validation cohort studies are required to evaluate the predictive performance of this model for clinical use.

## Author Contributions

**Kei Ito:** writing – original draft, formal analysis, methodology. **Akira Endo:** supervision, writing – review and editing. **Hiromasa Hoshi:** conceptualization, supervision, data curation. **Koji Ito:** data curation, supervision. **Tomohiro Akutsu:** supervision, data curation. **Hikaru Odera:** data curation, supervision. **Hideto Shiraki:** supervision, data curation. **Takeshi Yokoyama:** data curation, supervision. **Yasukazu Narita:** supervision, data curation. **Taro Masuda:** supervision, data curation. **Akira Suekane:** data curation, supervision. **Shigeru Yamagishi:** supervision. **Koji Morishita:** supervision.

## Ethics Statement

This study was approved by the Institutional Review Board of Fujisawa City Hospital (M2019‐018).

## Conflicts of Interest

The authors declare no conflicts of interest.

## Supporting information


**FIGURE S1:** The receiver operating characteristic curves for model performance stratified by perforation site.

**TABLE S1:** The baseline characteristics of patients died after surgery.

## Data Availability

Data are available from the corresponding author on reasonable request.
