# Supplementary figures and images for: Development of a Scoring System to Predict the Treatment Success for Nonoperative Management of Peptic Ulcer Perforation: A Secondary Data Analysis of PPAP Study
Source: Ann Gastroenterol Surg. 2025 Aug 12;10(1):95–102. doi: 10.1002/ags3.70074 (PMC12757152; doi:10.1002/ags3.70074)

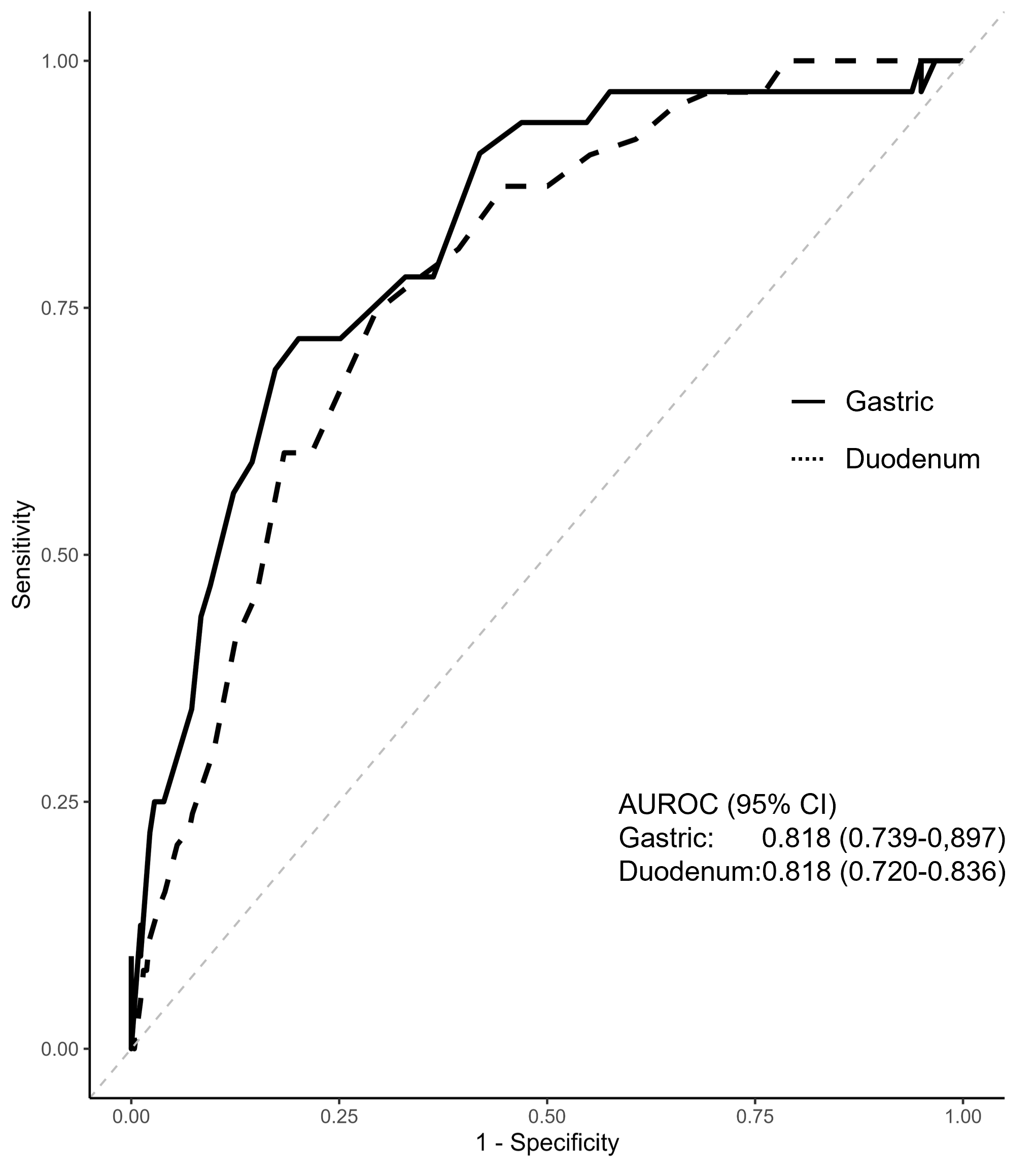

Supplement: Supplementary file 1 — FIGURE S1: The receiver operating characteristic curves for model performance stratified by perforation site. [file AGS3-10-95-s001.tif]
