# Supplementary material for: Development of a Scoring System to Predict the Treatment Success for Nonoperative Management of Peptic Ulcer Perforation: A Secondary Data Analysis of PPAP Study
Source: Ann Gastroenterol Surg. 2025 Aug 12;10(1):95–102. doi: 10.1002/ags3.70074 (PMC12757152; doi:10.1002/ags3.70074)
Supplement: Supplementary file 2 — TABLE S1: The baseline characteristics of patients died after surgery. [file AGS3-10-95-s002.docx]

| **TableS1. The baseline characteristics of patients died after surgery** | |
| --- | --- |
| **Characteristic** | **N = 25**^1^ |
| Sex, female | 13 (52%) |
| Age | 77 (71, 81) |
| Non-steroidal anti-inflammatory drugs | 8 (32%) |
| Steroid | 3 (12%) |
| Charlson comorbidity index | 7 (5, 10) |
| Peritoneal irritation signs |  |
| None | 6 (24%) |
| Upper abdomen only | 7 (28%) |
| Wider than upper abdomen | 12 (48%) |
| Ascites in CT |  |
| None | 2 (8.0%) |
| Upper abdomen only | 2 (8.0%) |
| Wider than upper abdomen | 21 (84%) |
| Body temperature, ℃ | 36.40 (36.00, 36.70) |
| Systolic blood pressure, mmHg | 111 (102, 132) |
| Heart Rate, bpm | 96 (89, 117) |
| White blood cell count, /μL | 8,000 (6,590, 14,100) |
| C reactive protein, mg/dL | 12 (7, 22) |
| Hemoglobin, mg/dL | 10.50 (9.00, 13.10) |
| Albumin, g/dL | 2.20 (2.00, 2.60) |
| Serum Creatinine, mg/dL | 1.53 (0.88, 2.40) |
| Sepsis |  |
| None | 13 (52%) |
| Sepsis | 6 (24%) |
| Septic shock | 6 (24%) |
| Time from onset to hospital visit, hours |  |
| Less than 6 | 7 (28%) |
| 6–12 | 5 (20%) |
| 12–24 | 3 (12%) |
| More than 24 | 10 (40%) |
| Perforated area |  |
| Anterior wall of the stomach | 4 (16%) |
| Posterior wall of the stomach | 3 (12%) |
| Anterior wall of the duodenum | 18 (72%) |
| Perforation diameter | 15 (5, 20) |
| Unknown | 2 |
| Hospital length of stay, days | 17 (4, 39) |
| ^1^n (%); Median (Q1, Q3)  The definition of sepsis and septic shock is based on the Sepsis-3 criteria.  Abbreviations: CT =computed tomography | |
